# Seismic Retrofitting of Existing Industrial Steel Buildings: A Case-Study

**DOI:** 10.3390/ma15093276

**Published:** 2022-05-03

**Authors:** Roberto Tartaglia, Aldo Milone, Alessandro Prota, Raffaele Landolfo

**Affiliations:** Department of Structures for Engineering and Architecture, University of Naples Federico II, Via Forno Vecchio 36, 80134 Naples, Italy; roberto.tartaglia@unina.it (R.T.); aldo.milone@unina.it (A.M.); alessandro.prota@unina.it (A.P.)

**Keywords:** existing structures, steel constructions, seismic retrofitting, finite element analyses

## Abstract

Industrial single-storey buildings are the most diffuse typology of steel construction located in Italy. Most of these existing buildings were erected prior to the enforcement of adequate seismic provisions; hence, crucial attention is paid nowadays to the design of low-impact retrofit interventions which can restore a proper structural performance without interrupting productive activities. Within this framework, an existing industrial single-storey steel building located in Nusco (Italy) is selected in this paper as a case-study. The structure, which features moment resisting (MR) truss frames in both directions, is highly deformable and presents undersized MR bolted connections. Structural performance of the case-study was assessed by means of both global and local refined numerical analyses. As expected, the inadequate performance of connections, which fail due to brittle mechanisms, detrimentally affects the global response of the structure both in terms of lateral stiffness and resistance. This effect was accounted for in global analyses by means of properly calibrated non-linear links. Thus, both local and global retrofit interventions were designed and numerically investigated. Namely, lower chord connections were strengthened by means of rib stiffeners and additional rows of M20 10.9 bolts, whereas concentrically braced frames (CBFs) were placed on both directions’ facades. Designed interventions proved to be effective for the full structural retrofitting against both seismic and wind actions without limiting building accessibility.

## 1. Introduction

Industrial single-storey buildings represent the majority of existing steel constructions located in Italy [1]. This kind of structural type mainly spread during the second half of the 20th century due to its capability of covering relatively large spans without recurring to complex technological solutions, and with affordable costs [2].

Hence, most Italian industrial steel buildings realised between the 1980s–1990s were designed in compliance with the CNR 10,011 [3] code, and, in particular, the vertical and the wind actions were accounted for as prescribed by CS.LL.PP. n. 56 and n. 140, respectively [4,5]. Only in 1986, the document Decreto Ministeriale 24 January 1986 [6] introduced the equivalent static forces to account for the seismic action. However, this code did not provide adequate instructions to ensure a satisfactory seismic performance, since it does not provide adequate prescription for the design of the local detailing. For instance, in the design of connections, no distinctions were made between ductile and brittle mechanisms. Moreover, as highlighted by [7,8], the lack of prescriptions for joints characterization often led to non-conservative design assumptions (e.g., in case of base connections).

Inadequate technical practices were also fostered by the common idea that seismic action could not govern design choices for industrial single-storey buildings, owing to their relatively low mass with respect to enclosed surfaces [2], similarly with what was observed for moment-resisting frames equipped with truss beams [9,10].

However, the occurrence of multiple seismic events, e.g., Friuli (1976), L’Aquila (2009), and Emilia (2012) earthquakes, proved the incorrectness of this belief, as several industrial buildings reported moderate-to-severe damages, with some relevant cases of global collapse also [11].

Differently from residential buildings, an important aspect that should be accounted for when dealing with industrial constructions is represented by indirect costs, i.e., expenses due to interruption of the productive activities for long time [12]. Namely, on most occasions, seismic damage to Italian industrial steel buildings consisted of local failures of connections, claddings, and/or roofing [7], though a few notable cases of global collapse occurred as well [13].

In light of these events, the interest in assessing and enhancing the seismic performance of steel structures [14,15,16,17,18] and, in particular, the industrial single-storey buildings has quickly developed up to present time. In particular, crucial attention is currently paid to the design of low-impact retrofitting interventions which simultaneously minimise time needed to resume productive activities and effectively prevent not only structural damage, but also damage on industrial machineries [19]. It is worth reporting that some contributions aiming at investigating the efficiency of low-impact retrofitting strategies on industrial buildings are already available in the literature. Formisano et al. [19] investigated the efficiency of global retrofitting interventions on an industrial steel structure located in Italy. The authors proved the effectiveness of concentrically braced frames (CBFs) with both X-shaped and portal (i.e., double Y-shaped) configurations in enhancing both strength and stiffness of the structure without preventing building accessibility. Hirde et al. [20] inspected the effectiveness of two retrofit strategies for a damaged industrial steel building located in India. Namely, a first low-impact seismic enhancement was achieved by welding new angle profiles to existing structural members (i.e., back-to-back). The authors investigated a more invasive solution involving the introduction of new truss beams below the existing load-bearing gables. Although being highly effective in improving both resistance and lateral stiffness of the structure, it should be remarked that this intervention was only feasible since no requirements about the minimum net height of the industrial building had to be fulfilled. Finally, Bournas et al. [21] analysed damages in industrial buildings (both with steel and precast RC structure) affected by the Emilia earthquake. On the basis of detected criticalities, the authors suggested that local interventions on beam-to-column joints and claddings connections could highly improve the performance of industrial buildings in seismic zones. The authors also highlighted the current absence of normative guidelines for the design and check of this kind of intervention.

Within this framework, the aim of this work is to design and check the effectiveness of low-impact retrofit interventions to increase the industrial building capacity against lateral action, without interrupting the building functionality. Indeed, this work is part of a wider Italian research project (Reluis WP5 [22]) aiming at verifying the validity of low-impact strategies for the seismic retrofitting of existing non-code-conforming buildings.

For this purpose, an existing single-storey steel building located in Nusco (Italy) is selected as a case-study; the structure was designed and erected during the 90 s in compliance with normative provisions enforced at the time [3]. 

Preliminary Finite Element Analyses (FEAs) on the selected case-study showed how the existing structure is rather deformable in both the principal directions, and unable to properly resist seismic actions. Therefore, both local and global retrofitting interventions were designed and verified by means of refined numerical models.

Indeed, one of the main aims of this paper is to underline the importance of the local behaviour of the steel joints in the assessment of existing structures, and how their performance should be accounted for also in the global analyses, since they could affect not only the local resistance, but also the lateral stiffness of the whole structure.

The paper is mainly divided into five sections: in the first part, the main features of the investigated case-study are presented. Concept and design procedures for low-impact seismic retrofit interventions are discussed in the second section, whereas in the third part, the main finite element (FE) modelling assumptions are summarised. The global and local seismic performance of the existing structure is presented in the fourth section, and finally, the efficiency of designed retrofit solutions is discussed in the last part. 

### 1.1. General Description of the Structure of the Selected Case-Study

The selected case-study is a single-storey industrial steel building that serves as a warehouse for an adjacent building in which aluminium products are manufactured. The dynamic response of the investigated structure, which was built later with respect to the production unit (i.e., between 1992 and 1999), was decoupled from the main building by means of a seismic joint.

**Figure 1:**
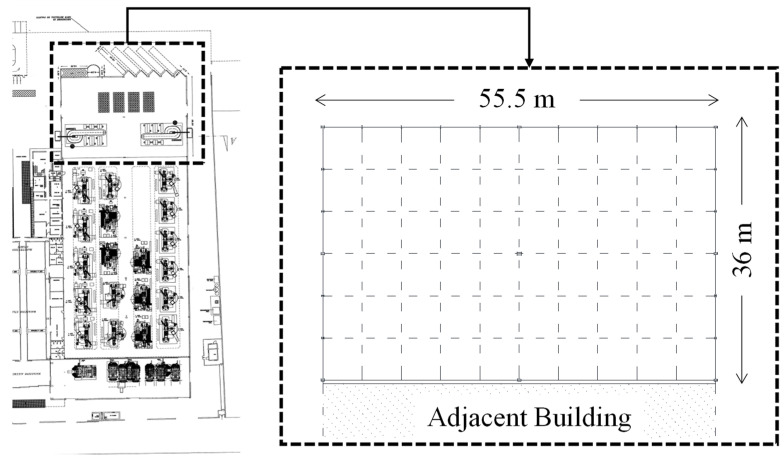
Geometrical features of the selected building according to the original design report.

Original design report and drawings, as well as on-site surveys, allowed the complete characterization of geometrical features of the selected building (see Figure 1). 

**Figure 2:**
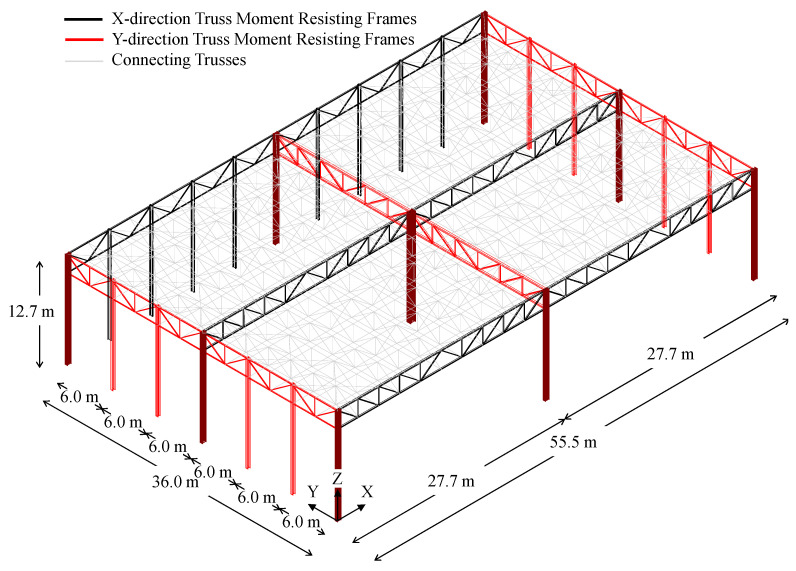
Geometrical features of the selected case-study and disposition of resisting systems in both directions.

The structure has a rectangular plan extending for 55.5 m in the longitudinal direction, and for 36 m in the transversal one; the total height of the building is equal to 12.7 m (see Figure 2).

Truss frames are used in X- and Y-directions to resist both gravity loads and horizontal actions. Both top and bottom chords are connected to the supporting columns, which are continuous in correspondence of the connections, thus creating a moment-resisting frame. Namely, three moment-resisting frames (MRFs) were placed in both X- and Y-directions, spaced out by intermediate connecting trusses (see Figure 2). 

Columns belonging to MRFs were made by means of welded hollow members; on the contrary, hot-rolled profiles (i.e., IPE 360 and HE 300B) were adopted for the claddings support system. Notably, all hollow columns are oriented with their strong axis being parallel to the Y-direction.

Coupled angle members having different cross-sections were adopted for seismic-resistant trusses, whereas both single and coupled angles were used for connecting trusses. 

The truss members are connected to each other and with columns by means of gusset plates placed within the gaps of back-to-back profiles, which are, in turn, bolted (in X-direction) or welded (in Y-direction) to column ends. Base connections were realised with extended stiffened plates in both directions.

According to the original design report, S235 grade steel was used for all members and plates, whereas 6.8 strength class bolts were adopted for the connections.

In light of the retrieved information, the highest level of knowledge (“KL3”—exhaustive knowledge) was attained for the selected case-study according to Italian provisions for existing buildings [23,24]. Hence, characteristic values of material properties were used for seismic analyses accounting for no reduction (i.e., a partial safety factor FC = 1 is assumed in [23,24] for KL3).

### 1.2. Description of Investigated Connections

**Figure 3:**
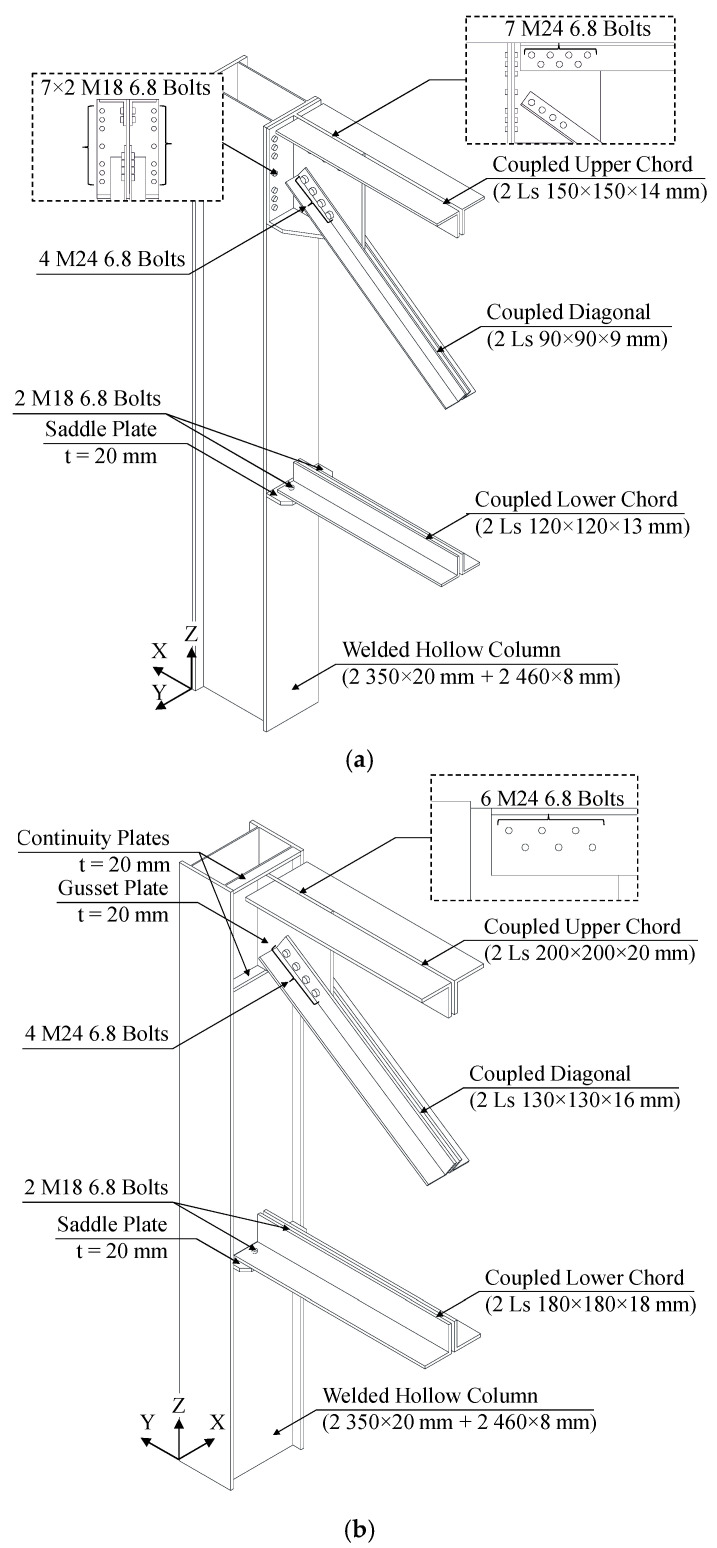
Details of truss-to-column connections adopted for MRFs: (**a**) X-direction and (**b**) Y-direction.

The main geometrical features of moment-resisting connections between truss members and columns are depicted in Figure 3. Owing to the constant orientation of all hollow columns, two different joint configurations were adopted in the X- and Y-direction.

A T-shaped 20 mm gusset plate is used to connect both the upper chord and the diagonal to the column (see Figure 3a) in the X-direction. Seven staggered M24 bolts are used for the upper coupled angles (2-Ls 150 mm × 150 mm × 14 mm), whereas four in-line M24 bolts are adopted for the coupled diagonals (2-Ls 90 mm × 90 mm × 9 mm). The gusset plate terminates with an end-plate, which is, in turn, bolted to the column flange (350 mm × 20 mm, welded to two 460 mm × 8 mm webs) by means of seven rows of M24 bolts. Moreover, the connection is further stiffened by means of two trapezoidal 20 mm ribs, placed at the base of the gusset plate. 

Contrariwise, a simpler configuration is adopted for the lower connection. Indeed, coupled members of the lower chord (2-Ls 120 mm × 120 mm × 13 mm) are connected with a single row of M18 bolts to a 20 mm saddle plate, which is welded to the column flange.

Slightly different solutions were adopted in the Y-direction due to the presence of the column web. Namely, the upper 20 mm gusset plate is directly welded to the web, which is locally stiffened by means of two 20 mm continuity plates (CPs).

Notably, the same kind and number of bolts used in the X-direction are adopted to connect the diagonal (2-Ls 130 mm × 130 mm × 16 mm) to the gusset plate, in spite of the different profiles employed, whereas only six M24 staggered bolts are used in this case to connect the upper chord (2-Ls 200 mm × 200 mm × 20 mm). Finally, the lower connection is almost identical to the X-direction one, aside from the 20 mm saddle plate being welded to both column web and flanges. Moreover, in this case, a single row of M18 bolts is used to connect coupled profiles of the lower chord to the saddle (2-Ls 180 mm × 180 mm × 18 mm).

## 2. Design Philosophy of Retrofit Interventions

The selected case-study shows poor seismic behaviour due to excessive lateral deformability and inadequacy of adopted structural details. Indeed, the preliminary analyses performed on a simplified model resulted in a very large first vibration period (T_1_ = 2.08 s, flexural mode in the Y-direction) and torsional deformability. Moreover, the moment-resisting (MR) joints in both X- and Y-directions showed local shortages in terms of elastic stiffness, resistance, and ductility, as will be shown in the next Sections.

The existing structural lateral deformability was checked in case of both seismic and wind actions at service limit sates (SLS) in accordance with the limitations provided by EN1998:1 [25] and EN1993:1-1 [26], respectively. Namely, for the seismic Damage Limitation (DL, return period of 50 years) limit state, a maximum lateral displacement capacity equal to 1/200 (0.5%) of the building height was assumed in compliance with EN1998:1 [25]. Contrariwise, wind loads, which were defined considering a rare load combination, were checked in terms of maximum lateral displacements at the top of the columns. For this purpose, a maximum displacement capacity equal to 1/300 of the column’s height was considered in compliance with EN1993:1-1 [26] prescriptions.

Both local and global retrofit interventions had to be designed for the investigated case-study; among the different possibilities [27,28,29,30], non-invasive retrofit interventions were conceived and designed in order to achieve a satisfactory seismic performance of the building without interrupting the productive activities.

Therefore, as will be presented in the next Section, concentric braced frames (CBFs) were introduced on the external perimeter of the existing building; moreover, the local performance of the MR joints was investigated by means of FEAs, and retrofit interventions were properly designed.

### 2.1. Design of Global Retrofitting Interventions

The design of global strengthening for the selected case-study was performed aiming at a full retrofit against seismic actions and wind loads.

Thus, the interventions were designed based on combined results from global and local FEAs. A first global assessment of the existing structure behaviour was conducted by means of static non-linear analyses; hence, pushover curves were simplified according to the N2 method [31], which allows deriving bi-linear equivalent force-displacement curves. Thus, the smooth pushover curves were converted into equivalent curves by equating the ultimate displacements (i.e., displacements corresponding to 80% of the maximum base shear measured on the degrading branch of the curves) and the areas underneath the force-displacement curves. According to EN1998:3—Annex B [31] prescriptions, the bi-linear pushover curves were interrupted when the maximum allowable plastic rotation was reached in the most stressed plastic hinge. 

**Figure 4:**
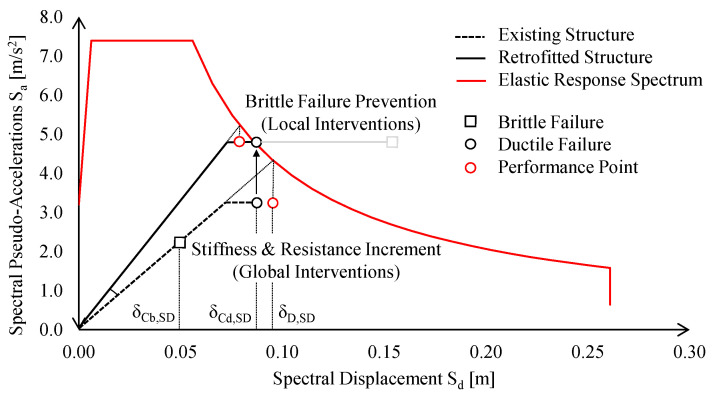
Graphical interpretation in ADRS domain for the design procedure of global retrofitting interventions.

The structural capacity was compared with the seismic demand at significant damage (SD) limit state (LS), transposing the bi-linear curves into an Accelerations–Displacements Response Spectrum (ADRS) domain. Therefore, the seismic demand on the structure, i.e., the so-called “performance point” (PP), was conventionally derived (see Figure 4, red hollow circle).

This procedure allows to assess the structural ductile capacity, disregarding brittle failure mechanisms (e.g., brittle bolts’ shear failure) that should be subsequently assessed. The second step involved the assessment of the local behaviour of the MR joints; in particular, as introduced in Section 3, a shortage was observed in the bottom part of the external MR joints. The real joints’ behaviour was investigated by means of both an analytical method and a refined finite element model; finally, its behaviour was accounted for in a new set of global analyses by introducing non-linear links in correspondence of the MR joints. 

The global retrofit intervention was ensured increasing the existing structure lateral stiffness and resistance; thus, the required stiffness increment was derived assuming the occurrence of ductile failure of the structure at the intersection with elastic response spectrum (ERS) as follows:

(1) $$\Delta K_{CBFs}\;=\;\frac{M_{TOT}\cdot S_{a,ERS}\left(S_{d,DF}\right)}{S_{d,DF}}\;-\;K_{ex}$$

where Δ*K_CBFs_* is the minimum lateral stiffness increment to be provided by new CBFs; *M_TOT_* is the total seismic mass of the structure; *S_a,ERS_*(*δ_Cd,SD_*) is the spectral pseudo-acceleration derived from the ERS for a spectral displacement equal to the *δ_Cd,SD_*, i.e., the spectral displacement corresponding to ductile failure of the existing structure; and *K_ex_* is the lateral stiffness of the existing structure.

Fulfilment of Equation (1) ensures that PP is compatible with the seismic response of the retrofitted structure provided that brittle failures are prevented. This additional requirement was achieved by means of local retrofit interventions, which will be discussed in detail in the following subsection.

The retrofit intervention was designed not only to satisfy seismic requirements, but also to verify the structural deformability against wind loads. Therefore, the minimum increase of lateral stiffness also accounted for lateral deformation limits introduced by EN1993:1-1 [26]. Namely, according to [26], maximum lateral displacements due to the wind loads should be smaller than 1/300 of the element’s length.

In order to account for both seismic and wind lateral stiffness requirements, Equation (1) becomes:

(2) $$\begin{array}{c} \Delta K_{CBF}\;=\;Max\;\left(\Delta K_{CBF-s};\Delta K_{CBF-w}\right)\\ \Delta K_{CBF}\;=\;Max\left(\;\frac{M_{TOT}\cdot S_{a,ERS}\left(S_{d,DF}\right)}{S_{d,DF}}\;-\;K_{ex};\;\frac{300\;F_{w,Ed}}{H_{c}}\;-\;K_{ex}\right) \end{array}$$

With *F_w,Ed_* being the design wind action acting in a given direction, and *H_c_* being the height of welded hollow columns.

It should be remarked that design criteria provided by Equations (1) and (2) hold true under the assumption of an in-plane rigid storey, which was granted by the presence of roof braces.

Finally, after determining minimum cross sections of braces accordingly, resistance and stability checks were performed on new CBFs for gravity, seismic, and wind load combinations as follows:

(3) $$N_{Ed,g,i}^{-}\;\leq \;N_{b,Rd,i}$$

(4) $$N_{Ed,E,i}^{-}\;\leq \;N_{b,Rd,i}\cup^{}\;N_{Ed,E,i}^{+}\leq N_{pl,Rd,i}$$

(5) $$N_{Ed,w,i}^{-}\leq N_{b,Rd,i}\cup^{}\;N_{Ed,w,i}^{+}\leq N_{pl,Rd,i}$$

where *N_Ed,g,i_*, *N_Ed,E,i_*, *N_Ed,w,i_* are the design axial forces in new CBF members due to gravity, seismic, and wind actions, respectively, whereas *N_b,Rd,i_*, *N_pl,Rd,i_* are the design buckling and plastic resistances of the same members, respectively. For the sake of clarity, in Equations (3)–(5), the superscript “−” is related to compressive axial forces, whereas the superscript “+” is adopted for tensile axial forces.

**Figure 5:**
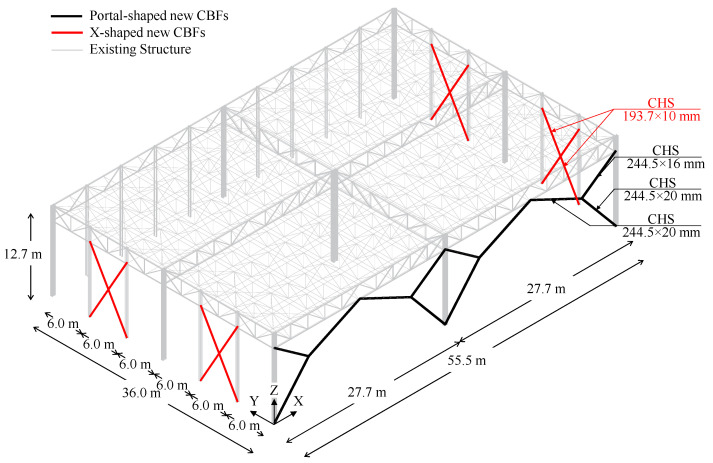
Description of adopted global retrofitting interventions.

Four X-shaped CBFs were placed along the Y-direction, according to design criteria reported in Equations (2)–(5), and CHS profiles (193.7 mm × 10 mm) were adopted. On the other hand, to minimise the footprint of new resisting systems, and to guarantee the passage of industrial machines as forklifts, two portal CBFs placed beside the facades were conceived in the X-direction. For this purpose, CHS profiles (244.5 mm × 20 mm and 244.5 mm × 16 mm) were properly selected according to the design criterion provided by Equation (1), and, hence, checked in terms of stability and resistance according to Equations (3)–(5) (see Figure 5).

It should be noted that the CBFs in both directions were designed in compliance with the last draft of the prEN1998-1-2 [32], currently under revision. According to [32], in the design of X-CBF, both tension and compression members of bracing systems should be considered in structural analysis, at the price of checking the possible occurrence of global instability phenomena under design compressive forces. This approach allowed a more appropriate evaluation of the actual lateral stiffness of the retrofitted building with respect to the only-tension members’ approach. However, the introduction of CBFs on the external perimeter of the existing structure implies an increase of the actions transferred to the foundation system; moreover, it should be noted that this type of intervention enables to increase both the lateral stiffness and the resistance of the existing structure, but it does not allow to increase its ultimate displacement capacity.

### 2.2. Design of Local Retrofitting Solutions

**Figure 6:**
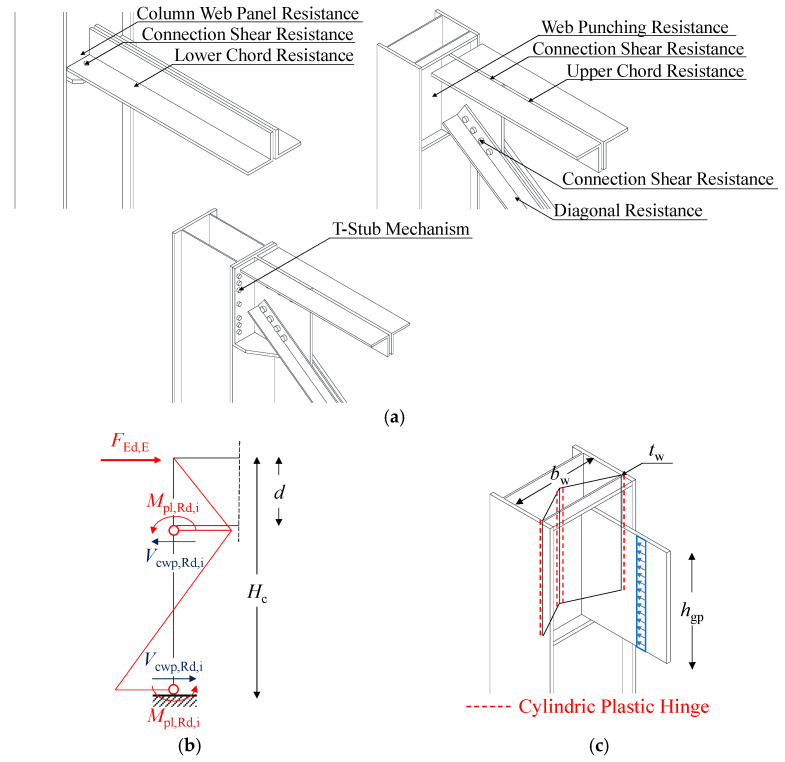
Considered failure mechanisms in MR truss connections for the design of local retrofitting interventions and assumed schemes for the estimation of resistance (**a**): column hinging (**b**) and web punching (**c**).

The design of local retrofitting solutions was performed in order to prevent local and premature brittle failures. For this purpose, multiple local collapse mechanisms were considered for MR truss connections in both directions (see Figure 6a), namely:

- Bolted connections shear resistance *F_con,Rd,i_* (due to bolt shearing, plate bearing, or net-area failure depending on the *i*-th connection configuration);
- Truss members axial resistance *N_truss,Rd,i_* (due to yielding in tension or buckling in compression depending on the considered *i*-th truss member);
- Column web panel (CWP) resistance *V_cwp,Rd,i_* (due to web shearing or column hinging);
- Upper connection resistance for other local mechanisms *F_up,Rd,i_* (due to T-stub opening in X-direction or web punching in Y-direction).

The design resistance of bolted connections was evaluated according to prescriptions from EN1993:1-8 [33], whereas usual formulations provided by EN1993:1-1 [26] were used to calculate the axial resistance of truss members and shear resistance of the column.

With regards to the column hinging mechanism, the equivalent resistance of the column was assumed equal to the shear force transmitted by the hollow profile in correspondence of the formation of two plastic hinges, i.e., at the column base and alongside the lower chord connection (see Figure 6b):

(6) $$V_{cwp,Rd,i}\;=\;\frac{2M_{pl,Rd,i}}{\left(H_{c}-d\right)}\;\;\;\;\;\left(column\;hinging\right)$$

where *M_pl,Rd,i_* is the plastic bending resistance of the column with respect to the *i*-th inflection axis, and *d* is the distance between centroids of the upper and the lower chord.

The T-stub opening mechanism in the X-direction was modelled according to provisions from [26], accounting for all possible failure modes (i.e., mode 1—pure plate yielding, mode 2—plate yielding + bolts tension failure, mode 3—pure bolts tension failure). 

With regards to web punching in the Y-direction, the resistance was estimated regarding the column web segment within the two CPs as a doubly-restrained plate subjected to a line load simulating the gusset plate contact force (see Figure 6c):

(7) $$F_{up,Rd,i}\;=\;\frac{2t_{w}^{2}h_{gp}f_{y}}{b_{w}}\;\;\;\;\;\left(column\;web\;punching\right)$$

with *t_w_* and *b_w_* being the column web thickness and width, respectively, and *h_gp_* being the gusset plate height.

The design of local retrofitting interventions was, hence, performed in order to achieve the hierarchy between ductile and brittle mechanisms for each considered connection. Namely, undesirable failure modes, such as bolts shearing, bolts tension failure, trusses net-area failure, or T-stub mode 3 collapse, were prevented by introducing new strengthening elements and/or improving existing connections with the aid of new high-strength bolts.

From analytical calculations, lower chord connections in both the X- and Y-direction resulted in the weakest component for existing MR truss joints, with a shear capacity equal to 183 kN. The corresponding maximum base shear *V_b,R_*, which can be approximately estimated using the a simplified structural scheme (i.e., similar to the one in Figure 6b), resulted equal to 74 kN.

**Figure 7:**
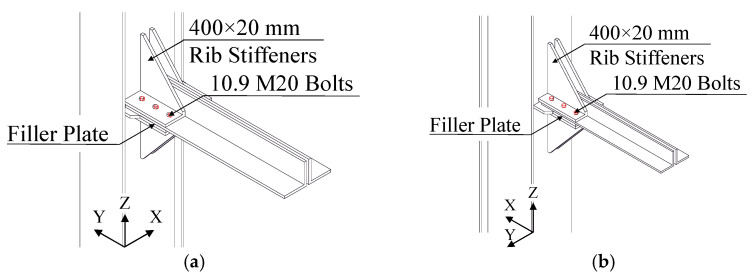
Local retrofit solutions on MR joints in both X- (**a**) and Y-directions (**b**).

Hence, local retrofit solutions were designed in order to obtain a stronger connection with a shear resistance larger than the axial capacity of the connected truss elements. Therefore, the retrofit solution involving the introduction of four 150 mm × 10 mm rib stiffeners was conceived for both directions, in order to: (i) increase connection stiffness, and (ii) increase the number of shear plans (from one to two). High-strength 10.9 pre-loaded M20 bolts were used in place of existing bolts. Moreover, new *Φ*21 holes were drilled in lower chord profiles to place two additional bolt rows in both directions (see Figure 7). The shear resistance of new connection results equal to 2 times the buckling resistance of the trusses element in the X-direction (2262 kN and 888 kN, respectively), whereas it results slightly higher than the buckling resistance of the trusses element in the Y-direction (2262 kN and 2222 kN, respectively).

## 3. Main Modelling Assumptions

### 3.1. Global Modelling of the Structure

**Figure 8:**
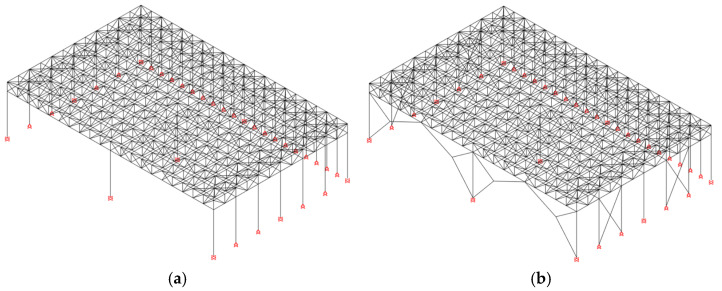
Global model main features: (**a**) Existing and (**b**) Retrofitted structure.

Two global finite element models (FEMs) of the entire structure were developed using Seismostruct 2022 [34] (see Figure 8). The first model was built in order to perform the global structural assessment of the industrial building disregarding the presence of the connections; thus, wire elements were adopted for beams and columns, modelled in correspondence of centroidal axes of steel profiles. The presence of horizontal X-braces, which ensure in-plane rigidity of the roof, was accounted for by means of a diaphragm constraint. Foundations and relative base connections were modelled by means of equivalent restraints; namely, the extended stiffened base plates allowed to model column-foundation connections as fixed restraints in both the X- and Y-direction. Connections among truss elements, which can be regarded as internal hinges, were modelled by means of local releases. In order to correctly account for the flexural continuity of both lower and upper chords, no releases were introduced in such elements.

Non-linear behaviour of the investigated members was accounted for by the introduction of lumped plastic hinges, which were defined according to prescriptions from ASCE-13 [35]. Namely, N-M_x_-M_y_ plastic hinges were adopted for the columns, i.e., at the base and in correspondence of the lower chord connection, in order to account for flexural response of hollow columns in both directions in presence of axial forces. On the other hand, non-symmetric axial hinges were introduced in truss elements to model both steel yielding in tension and possible global instability phenomena in compression.

The second global model was formally identical to the previous one, with the only exception of non-linear links, which were placed in correspondence of truss-to-column intersections to account for the local response of bolted connections. As will be shown in the next Section, the behaviour of links was calibrated against the results of local FEAs.

According to the original design report, the yielding strength *f_y_* of existing members was set equal to 235 N/mm^2^, whereas European S355 steel grade (*f_y_* = 355 N/mm^2^) was used for retrofitting interventions.

The presence of non-structural elements was accounted by means of equivalent area loads. Namely, a uniform load *g*_2*k*,*r*_ = 1.6 kN/m^2^ was assumed for the roofing system (i.e., composed by steel sheeting + isolating layer + ballast), whereas a unitary weight *g*_2*k*,*c*_ = 0.1 kN/m^2^ was considered for lightweight claddings. Moreover, live loads due to snow (*q_sk_*) and roof maintenance (*q_rm_*) were also introduced according to Italian normative provisions in force [23,24]. In particular, *q_sk_* = 3.1 kN/m^2^ was considered, owing to the high altitude of the construction site (≈1000 m a.s.l.), whereas *q_rm_* was set equal to 0.5 kN/m^2^.

Static non-linear analyses (SNLAs) were performed on global FEMs according to prescriptions from EN1998:3 [31]. Namely, a maximum inter-storey drift (ISD) equal to 6% was imposed in both directions, assuming the roof centre of masses as the control point. 

### 3.2. Local Modelling of the Truss MR Joints

**Figure 9:**
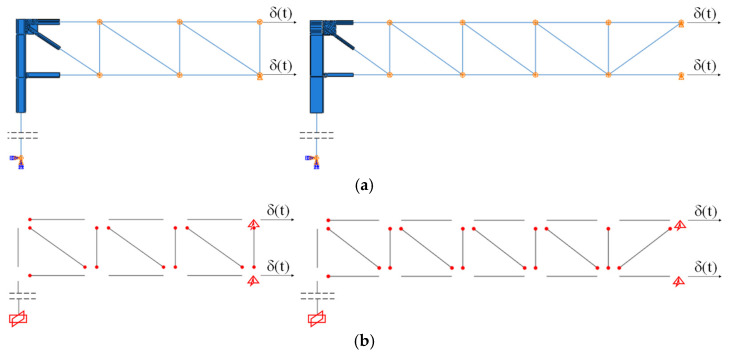
Local FEMs main features: (**a**) Abaqus local Modelling and (**b**) Sesimostruct local Modelling (Sub-assemblies).

Refined FEMs of truss MR joints were developed using ABAQUS 6.13 [36]. FEAs were performed considering a sub-assemblage of the whole structure, which is obtained by extracting the truss in correspondence of the inflection point of axial force diagrams in the chords under horizontal actions, i.e., at the chord midspan. Structural continuity was, hence, restored by means of proper boundary conditions (see Figure 9a).

In order to simulate the structural response of the truss MR frame under lateral loads, both monotonic and cyclic horizontal displacement histories were applied at the chord ends. Namely, a peak ISD equal to 6% was reached in monotonic FEAs, whereas AISC 341 loading protocol [37] was used for cyclic analyses, with a maximum ISD equal to 4%. 

In order to balance computational effort with analyses accuracy, only the upper end of the column, the connections, and the ends of truss members were modelled by means of solid elements, whereas wire elements were adopted for all other parts. Therefore, rigid MPC constraints were introduced at the interface among wire and solid instances. 

Experimental tests were not conducted on the investigated MR truss joints; therefore, the numerical modelling assumptions were set consistently with the ones adopted by the authors in previous research [38,39], and validated against experimental tests on beam-to-column steel joints. In particular, all solid parts were discretised using C3D8R solid element type (i.e., 8-node linear brick, reduced integration), whereas B31 beam elements (i.e., 2-node linear beams) were adopted for wire parts. The mesh density was defined on the basis of results from sensitivity analyses reported in [40,41]. In particular, a mesh size equal to 20 mm was set for beams and columns, whereas bolts and plates were discretised by means of a 5 mm mesh, with at least two elements through the thickness. 

The Von Mises criterion was used to model steel yielding, and both kinematic and isotropic hardening were accounted for by means of material parameters provided by [42].

In compliance with global FEMs, yielding strength of existing profiles and plates was set equal to 235 N/mm^2^, whereas yielding *f_yb_* and ultimate tensile strength *f_tb_* of 6.8 class bolts were assumed equal to 480 and 600 N/mm^2^, respectively. With regards to stiffening elements, *f_y_* = 355 N/mm^2^ was considered. Moreover, 10.9 class high-strength bolts were adopted for the seismic retrofit (*f_yb_* = 900 N/mm^2^, *f_tb_* = 1000 N/mm^2^).

Bolt clamping was simulated by means of the “Bolt Load” command. In order to account for the long service life and the absence of a controlled pre-loading, a low clamping stress equal to 0.35 *f_tb_* was considered for existing bolts. Contrariwise, a clamping stress equal to 0.7 *f_tb_* was adopted for new high-strength bolts according to provisions from EN1993:1-8 [33].

“Surface-to-Surface” interactions were introduced to model contact among the elements. Namely, a “Hard contact” formulation was used for normal contact behaviour, whereas a “Penalty” formulation was considered for tangential behaviour, with the friction coefficient being equal to 0.3. Finally, continuity among welded parts was modelled by means of “Tie” constraints.

As anticipated, the local FEAs results will be directly accounted for in the global analyses of the whole industrial building by the introduction of non-linear links placed in lower part of the MR truss joints. Figure 9b depicts the local Seismostruct [34] model having the same geometrical and mechanical features of the ABAQUS model, adopted for the calibration of the non-linear links.

A multilinear curve [34] was adopted for modelling the non-linear links behaviour under both monotonic and cyclic actions.

## 4. Performance of the Existing Structure

### 4.1. Global Assessment

The existing building is located in Nusco (Italy), with a peak ground acceleration (PGA) equal to 0.238 g; a soil topography class “T1” and the stratigraphy class “C” were used according to geotechnical considerations drawn from the original design report.

**Figure 10:**
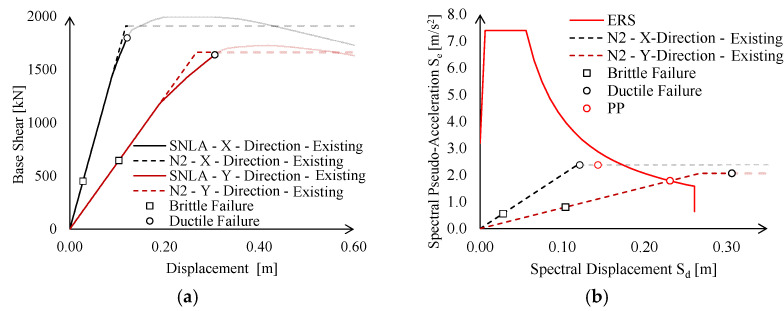
Static non-linear analyses (SLNA) of the existing building in both X- and Y-directions: (**a**) pushover curves and (**b**) ADRS domain checks.

According to both Italian and European codes [23,24,25], the seismic performance of the existing building at significant damage (SD) limit state (LS) was investigated by means of static non-linear (pushover) analyses (SNLA) (see Figure 10a). 

The pushover curves were approximated with equivalent elastic-plastic bi-linear curves, which were imported in the ADRS plan in conjunction with elastic response spectrum (ERS), defined through site-dependent seismic hazard maps adopted by Italian provisions [20] (see Figure 10b). 

The displacement demand at the SD limit state (*δ_D,SD_*) was compared against the brittle (*δ_Cb,SD_*) and ductile (*δ_Cd,SD_*) displacement capacity according to N2 method. In particular, for the investigated case-study, the brittle failure corresponds to the collapse of shear connections between the trusses and the column, whereas the ductile failure is governed by the central column that reaches its maximum rotation capacity.

The structural performances were checked also in terms of deformability against the seismic and wind actions. The seismic action was taken into account checking the Damage Limitation (DL) limit state for which the displacement demand (*δ_D,DL_*) was defined as done for the SD limit state, whereas the displacement capacity was assumed as 0.5% of the total height of the building (*δ_C,DL_*). On the other hand, wind action is represented by a simplified set of pressures according to the Italian code requirements [20,21]. Thus, the lateral displacements at the top of the columns were monitored and compared against horizontal displacement limits.

The analysis results are summarised in Table 1, where the structural lateral displacements at SD, DL, and in case of wind actions were pointed out and compared against the corresponding displacement capacity.

As it can be observed, the existing structure does not meet the required performance in terms of resistance and deformability when subjected to both seismic and wind actions.

### 4.2. Local Assesment

**Figure 11:**
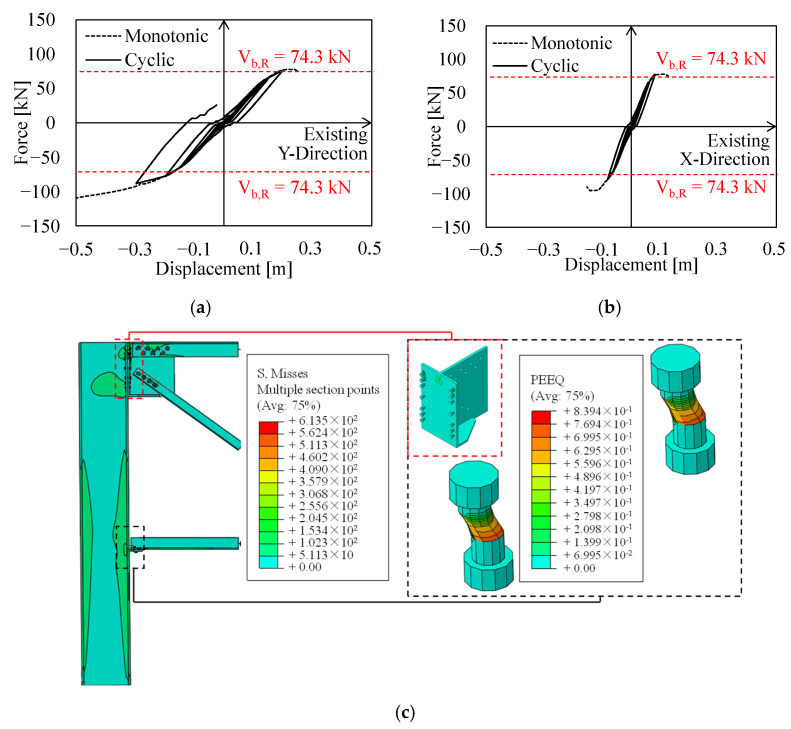

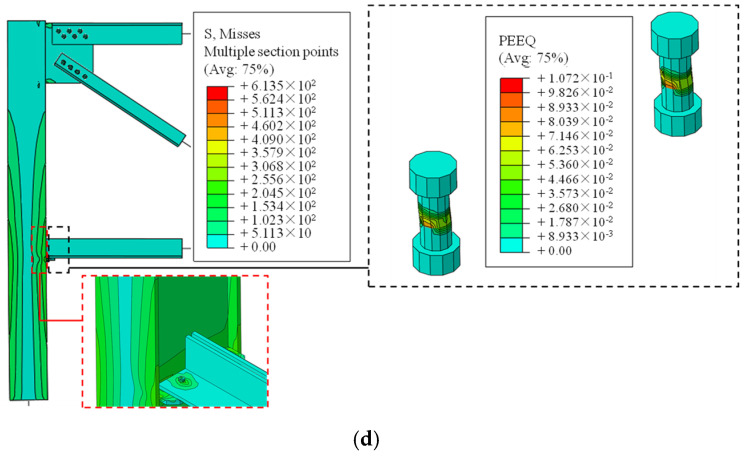
Local performance of existing MR truss connections in terms of base shear vs. displacement curves and distributions of MISES and PEEQ. (**a**) Base Shear vs. Displacements (X); (**b**) Base Shear—Displacements (Y); (**c**) Von Mises and PEEQ distribution of MR truss in X direction under hogging actions; (**d**) Von Mises and PEEQ distribution of MR truss in Y direction under hogging actions.

Figure 11 depicts the results of local analyses performed on both MR truss joints in terms of base shear-displacement curves, Von Mises stresses (MISES), and equivalent plastic strains (PEEQ). It can be noticed that the local seismic performance of truss joints in the X- and Y-direction is poor, owing to premature failure of lower chord connections in both cases; namely, existing M18 bolts (having a strength class equal to 6.8) fail in shear for rather low values of ISD (1–2%). 

Such undesirable failure mechanisms sensibly affect the cyclic performance of connections, which exhibits a significant pinching effect in both directions (see Figure 11a,b).

As expected, the ultimate displacement capacity of local assemblies under cyclic loadings is lower than the related capacity under monotonic lateral actions due to cyclic degradation of bolts. Moreover, since the same number and kind of bolts are used in both directions to connect lower chords (i.e., two M18 6.8 bolts), the peak base shear in both directions is basically identical (≈80 kN, +7% with respect to analytical calculations).

Contrariwise, a significant difference can be noticed in terms of both elastic stiffness and ultimate displacements, with the X-direction assembly being more rigid and having a displacement capacity which is about half of the related capacity in the Y-direction. 

This outcome clearly depends on the difference in lateral stiffness of truss frames located in the two orthogonal directions. Indeed, the X-direction truss frame is the most rigid resisting system, owing to the favourable orientation of the hollow column (i.e., inflected about its strong axis). 

Therefore, considerably higher actions are transferred by the bolts for the same value of ISD, resulting in a premature exceedance of the connection shear resistance.

Notably, the monotonic local behaviour of Y-direction MR connections is asymmetric with respect to deflections orientation (see Figure 11b, dashed curves). Indeed, though the Y-direction truss quickly fails for hogging deflections, the base shear transmitted in case of sagging deflections keeps increasing even for rather high values of ISD.

This depends on a secondary mechanism in which the lower chord in compression transfers axial forces by direct contact with the column web after bolts have exceeded their elastic range. On the contrary, contact load-bearing does not trigger in lower chord connection in the X-direction, due to the larger extension of the saddle plate, since bolt fracture occurs prior to chord-to-column contact.

With respect to local mechanisms in upper connections, PEEQ distribution in the X-direction (i.e., on the T-stub gusset plate) under sagging deflections confirms the activation of mode 2 failure, as foreseen with analytical models provided by EN1993:1-8 [33] (see Section 3.2). Indeed, PEEQ are spread among both the gusset and the bolts, resulting in a satisfying local ductility as mode 3 collapse is prevented (see Figure 11c).

Therefore, the analytical approach allows to adequately predict the shear resistance of the connections, but it is not able to account for the local mechanisms and the different stiffness of the two joints. Therefore, in order to account these aspects within the global model of the structure, non-linear links, properly calibrated against local FEAs results, were introduced.

**Figure 12:**
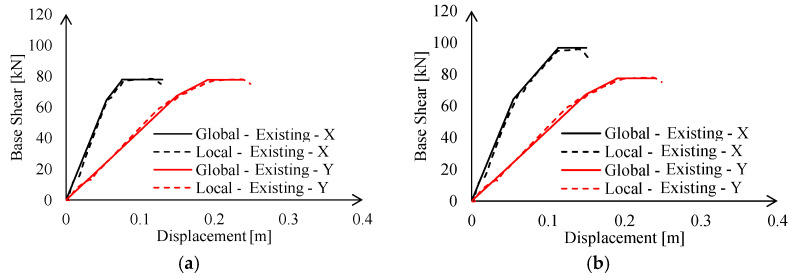
Calibration of the non-linear links of the existing MR connections under: (**a**) hogging and (**b**) sagging moment.

Results of the calibration procedure are reported in Figure 12 in terms of base shear force vs. imposed displacements. It can be observed that non-linear links are perfectly able to reproduce the local behaviour of the MR joints in term of elastic stiffness, resistance, and ultimate displacement capacity.

The local behaviour of MR truss joints affects the global behaviour of the entire structure. Indeed, a lateral stiffness reduction due to lower connection shortage can be noticed (see Table 2). This effect can be mostly appreciated in the X-direction (−6.7% with respect to the first set of global FEAs), i.e., the one in which stiffer frames are located. Hence, lower connection acts as an additional source of deformability in series with steel profiles; therefore, its effect becomes relevant in case of more rigid assemblies. Contrariwise, this effect is basically negligible in the Y-direction, i.e., for most deformable trusses.

As expected, the introduction of the non-linear links has a large influence on the local model behaviour, i.e., a variation of 18.6%. Contrariwise, the performance of the global structure is less affected by the presence of the link, as depicted in Table 2, in terms of elastic stiffness. This result mainly depends on the number of the connections where the non-links were introduced with respect to the total amount of joints.

**Figure 13:**
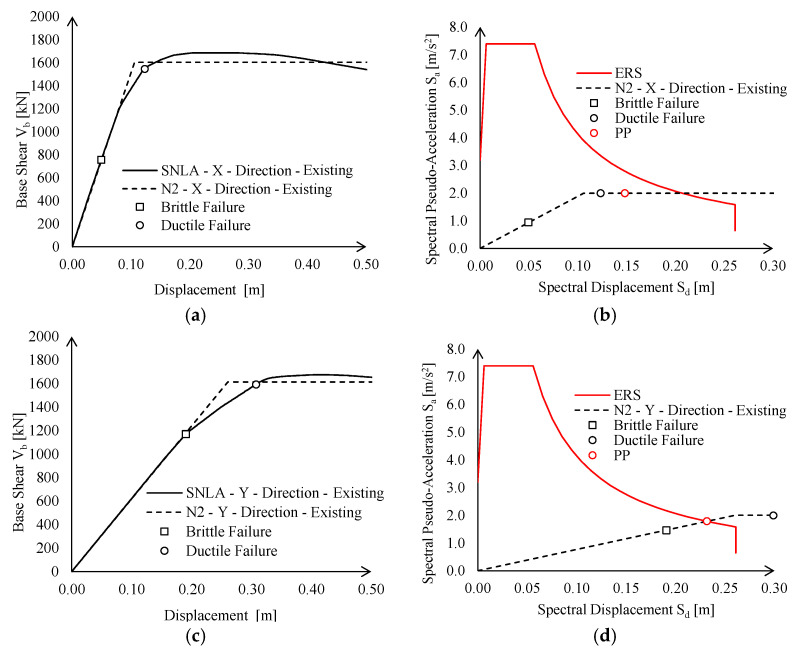
Global performance of existing structure in terms of pushover curves and ADRS domain checks according to EN1998:3 [31] provisions: (**a**) Pushover curves in X-direction, (**b**) ADRS domain checks in X-direction, (**c**) Pushover curves in Y-direction, (**d**) ADRS domain checks in Y-direction.

On the other hand, the introduction of non-linear links actually changes seismic demand on the construction, as PP is evaluated based on the lateral elastic stiffness of the structure. Global behaviour of the existing structure accounting for connection performance is summarised in Figure 13 in terms of pushover curves and in the ADRS domain. For the sake of clarity, in the following, smooth pushover curves are labelled as “SNLA”, whereas bi-linear equivalent curves derived according to the N2 method are labelled as “N2”. 

The existing structure does not attain a satisfying seismic performance either in the X- or Y-direction due to brittle failure of connections. Nevertheless, significant differences can be noticed with respect to the structural behaviour in the two directions, namely: 

In the X-direction, the seismic behaviour is inadequate, not only owing to local connection failures, but also in terms of global stiffness and resistance. Indeed, if local failures were prevented (i.e., by means of local retrofit interventions), the structure would still exhibit an insufficient displacement capacity (see Figure 13b, black circle), i.e., lower than the corresponding demand defined by PP (Figure 13b, red circle);

In the Y-direction, seismic checks in the ADRS domain are not fulfilled, only due to the brittle failure of lower chord connections. Indeed, PP is attained for a spectral displacement lower than the corresponding ultimate displacement (see Figure 13d).

Therefore, the disposition of new CBFs for global seismic enhancement was actually required only in the X-direction. Nevertheless, as expected, the existing structure results as highly deformable in the Y-direction. Therefore, CBFs should still be installed in this direction to fulfil deformability requirements for wind loads (see Equation (2)). Namely, the maximum lateral deflection of hollow columns in the Y-direction is equal to 0.13 m (see Table 1); hence, a stiffness increase equal to about 2 times *K_ext_* should be provided by new bracings.

## 5. Performance of the Retrofitted Structure

**Figure 14:**
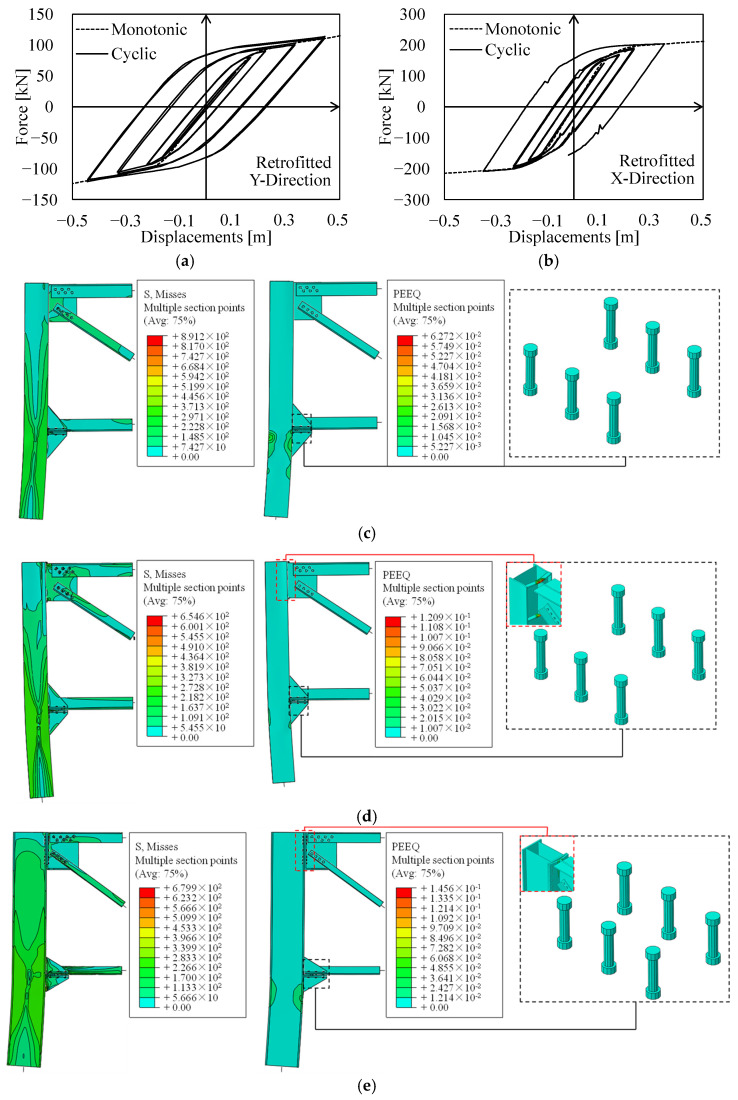

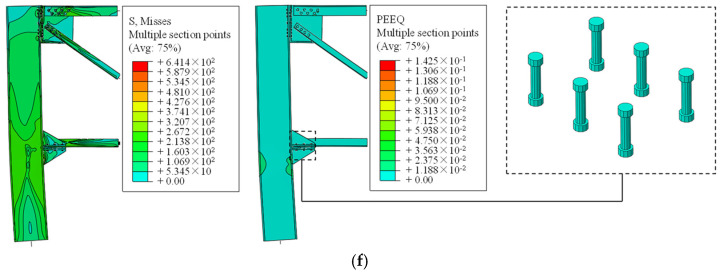
Local performance of retrofit interventions in terms of base shear vs. displacement curves and distributions of MISES and PEEQ. (**a**) Base Shear vs. Displacements (X); (**b**) Base Shear vs. Displacements (Y); (**c**,**d**) Von Mises and PEEQ distribution of MR truss in X direction under hogging and sagging actions; (**e**,**f**) Von Mises and PEEQ distribution of MR truss in Y direction under hogging and sagging actions.

The seismic performance of the retrofitted structure is, hence, reported both in terms of local response of enhanced MR truss connections and global performance of the retrofitted structure. Local behaviour of the two MR joints is depicted in Figure 14 in terms of base shear-displacement curves and distribution of Von Mises stresses (MISES) and equivalent plastic strains (PEEQ).

The retrofit intervention allows to effectively achieve satisfying seismic behaviour, as ductile mechanisms (i.e., column hinging) are promoted in place of brittle connection failures. Indeed, plastic strains are concentrated in hollow profiles at both the column base and lower chord intersection, whereas retrofitted connections always remain in their elastic range (see Figure 14c–f). The cyclic behaviour of both directions’ MR connections is positively affected by this condition, as hysteretic loops are sensibly wide and stable, allowing an efficient dissipation of seismic energy through the activation of plastic deformations within the columns.

It can also be noticed that there are some minor differences in terms of non-linear behaviour among monotonic and cyclic local FEAs. This outcome depends on cyclic hardening of the column base material, which results in higher transmitted shear force for smaller values of ISD with respect to monotonic conditions. 

**Figure 15:**
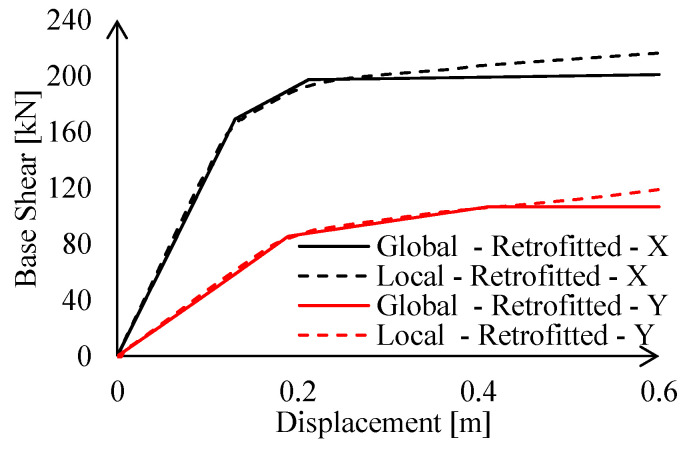
Results of the calibration procedure for retrofitted connections.

As done for the existing joints, the local performance of the MR joints was accounted for in the global analyses by means of properly calibrated non-linear links. Figure 15 depicts a very good agreement in terms of elastic stiffness, maximum resistance, and ultimate capacity between the FE results and the non-linear link behaviour. It should be observed that, due to the strengthening interventions, the MR joints have symmetric behaviour; this is the reason why, in Figure 15, only the response under sagging moment in both X and Y directions is depicted.

**Figure 16:**
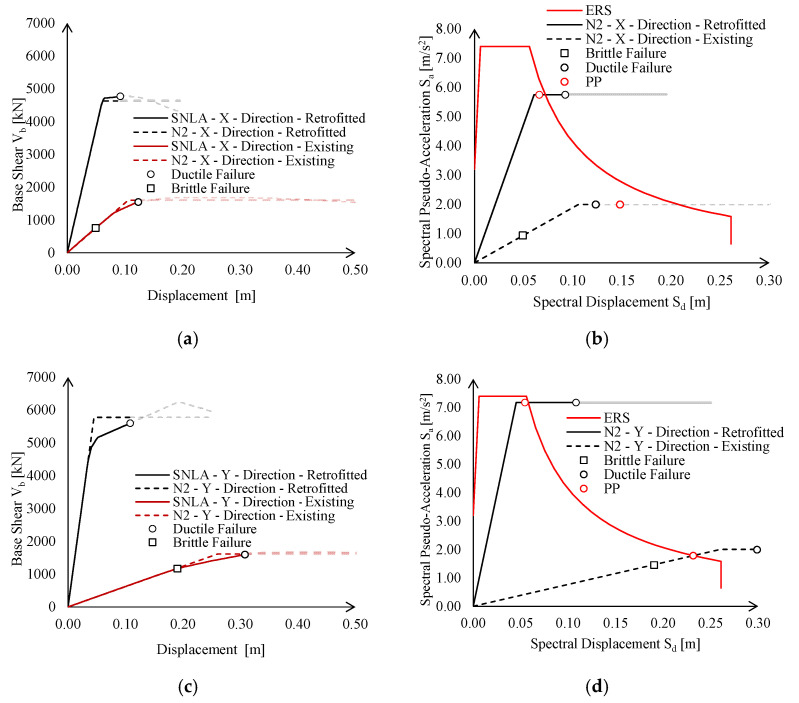
Global performance of existing structure in terms of pushover curves ((**a**,**c**) in X and Y directions respectively) and ADRS domain checks according to EN1998:3 [31] provisions ((**b**,**d**) in X and Y directions respectively).

Global behaviour of the retrofitted structure is summarised in Figure 16 in terms of pushover curves and in the ADRS domain.

The strengthening interventions allow to strongly increase the elastic stiffness and resistance of the existing structure up to a complete seismic retrofit. Moreover, lateral deformability checks for wind action are fulfilled with a significant safety margin (*δ_C_*/*δ_D_* is equal to 4 and 5.7 in X- and Y-direction, respectively—see Table 3). Indeed, with regards to the Y-direction, minimum cross-sections deriving from stiffness requirements (see Equation (2)) were enlarged to avoid global buckling of braces under gravity loads (see Equation (3)). Contrariwise, lateral deformability requirements for wind actions resulted as fulfilled in the X-direction due to the predominance of seismic action.

The pushover curves in both X- and Y-directions were stopped in correspondence of the ductile failure mechanism due to the diagonals in compression, which reach their maximum inelastic deformation capacity, defined as reported in [31]. Contrariwise, all the MR joints remain in their elastic range.

## 6. Conclusions

In the present paper, the effectiveness of low-impact seismic retrofitting interventions was investigated by means of global and local numerical analyses on a case-study of an existing industrial single-storey steel building located in Italy.

Particular attention was paid to the local failure modes and their influence on the global structural analyses; thus, refined numerical models were built to investigate the local MR truss joints behaviour. Their performances were successively accounted for in the global structural analyses by the introduction of non-linear links properly calibrated on the obtained FEAs results.

The investigated structure shows both local and global shortages; from the results of numerical analyses, the following conclusions can be pointed out:

- The investigated existing structure is very deformable in both the principal directions; showing excessive deflections under wind actions;
- The global structural behaviour is highly influenced by local deficiencies. Indeed, brittle failures always anticipate more ductile mechanisms, and lateral deformability is worsened by the lower stiffness of connections;
- The local seismic performance of MR truss joints in both the X- and Y-direction is poor due to premature failure of bolted connection among lower chords and hollow columns, for rather small values of ISD (1–2%);
- The hysteretic behaviour of joints is significantly affected by a pinching effect exhibited in both the principal directions;
- The introduced non-linear links are able to perfectly reproduce the local joint behaviour in term of elastic stiffness, resistance, and ultimate displacement, allowing to account for the real joint performance also in global FEAs;
- The real joint stiffness, evaluated by means of refined FEAs, and accounted for in the global analyses by the introduction of non-linear links, influences the whole structural behaviour, and should be properly accounted for in the existing structural assessment.

The global resistance and stiffness of the structure were increased by means of new CBFs in both directions, whereas the local performance of MR joints was enhanced by the introduction of 400 mm × 20 mm rib stiffeners and new rows of 10.9 M18 bolts.

From the numerical analyses results, the following conclusive remarks can be drawn:

- The design procedure adopted for the retrofitting of MR joints results in a very ductile mechanism under both monotonic and cyclic loads; the joints behave in elastic range up to 4% of rotation. For high rotations, the failure mode is governed by plastic deformations within the column and some local plasticity within the trusses and plates, whereas the bolts remain in elastic range;
- The global seismic performance of the retrofitted structure is positively influenced by local interventions that allow to ensure a ductile behaviour to the whole structure up to the formation of the plastic hinges in the columns;
- The introduction of the new CBFs in both directions allow to provide a sufficient elastic stiffness and resistance to the whole structures against both seismic and wind actions;
- The local and global retrofit interventions were designed to not interrupt the productive activities within the building, and to minimise the impact on the working spaces. Thus, the CBFs were designed to be placed on the external façade of the building, and their shape does not limit either the height or the required spaces for the access of industrial vehicles and machineries. Contrariwise, the local intervention should be performed in the inner part of the building, but their installation involves only a small portion of the entire structure.

## Figures and Tables

**Table 1:** Seismic and wind checks for the existing structure in terms of displacements.

Dir.	Significant Damage (SD)				Damage Limitation (DL)			Wind Action		
	*δ_D,SD_*	*δ_Cb,SD_*	*δ_Cd,SD_*	$\frac{\delta_{Cb,SD}}{\delta_{Dd,SD}}$	*δ_D,DL_*	*δ_C,DL_*	*δ_C,DL_*/*δ_D,DL_*	*δ_D_*	*δ_C_*	$\frac{\delta_{C}}{\delta_{D}}$
(-)	(m)	(m)	(m)	(-)	(m)	(m)	(-)	(m)	(m)	(-)
X	0.14	0.12	0.03	0.21	0.043	0.063	1.45	0.05	0.04	0.87
Y	0.23	0.30	0.10	0.43	0.059	0.063	1.05	0.13	0.04	0.32

**Table 2:** Results for the existing structure in terms of elastic stiffness evaluated accounting for/disregarding the local connection performance.

Dir.	Model	Elastic Stiffness		Variation
-	-	Without Links kN/m	With Links kN/m	-
X	Global	16,121.3	15,042.0	−6.7%
	Sub-assembly	1147.7	1178.1	−18.6%
Y	Global	6214.7	6195.2	−0.2%
	Sub-assembly	444.5	442.8	−0.5%

**Table 3:** Seismic and wind checks for the retrofitted structure in terms of displacements.

Dir.	Conf.	Significant Damage (SD)				Wind Action		
		*δ_D,SD_*	*δ_Cb,SD_*	*δ_Cd,SD_*	*δ_C,SD_*/*δ_Dd,SD_*	*δ_D_*	*δ_C_*	*δ_C_*/*δ_D_*
-	-	m	m	m	-	m	m	-
X	As Built	0.15	0.05	0.12	0.33	0.06	0.04	0.66
Y		0.23	0.19	0.31	0.61	0.16	0.04	0.25
X	Retrofitted	0.06	-	0.09	1.5	0.01	0.04	4
Y		0.05	-	0.11	2.2	0.007	0.04	5.7

## Data Availability

The data used in this study to support presented findings are included within the article.
